# Emergency Room Nurses’ Experiences in Person-Centred Care

**DOI:** 10.3390/nursrep12030045

**Published:** 2022-07-04

**Authors:** Jang Mi Kim, Na Geong Kim, Eun Nam Lee

**Affiliations:** 1 Nursing Department, Masan University, 2640 Gyeongsangnam-do, Masan 51217, Korea; rosekim@masan.ac.kr; 2 College of Nursing, Dong-A University, Busan 49201, Korea; nakami1006@naver.com

**Keywords:** emergency care, nurses, person-centred care, emergency department

## Abstract

Implementing person-centred care is often considered difficult in congested emergency rooms. The purpose of this study was to understand person-centred care experienced by emergency room nurses in depth and examine the essence of emergency room nurses’ lived experience of the person- centred care. Eight nurses working in the emergency room of a large hospital in South Korea and who had over six months of experience were surveyed via semi-structured interviews in February 2019. The data were transcribed and analysed using Colaizzi’s framework. The major findings related to person-centred care experiences among emergency room nurses were: (1) feeling distanced from patients; (2) guilt and frustration; (3) accepting patients’ symptoms and emotions as they are; (4) person-centred care as a domain of nursing that cannot be replaced by machines; and (5) nursing as an art wherein the minutest details make a difference. Providing person-centred practice in the emergency room is difficult, but it will not only improve the quality of patient care but also increase the job satisfaction of nurses. Based on an in-depth understanding of person-centred care experienced by emergency nurses, it will contribute to enhancing the quality of nursing care in the emergency room.

## 1. Introduction

Person-centred care involves respectful nursing care, considering the values and needs of each patient and accepting their decisions based on the understanding that patients are unique individuals [1,2]. Its goal is to maximise nursing efficiency by enhancing the quality of nursing and increasing both nurse and patient satisfaction [3].

Since the US Institute of Medicine [1] presented person-centredness as a core value in health policy-making, the World Health Organisation has defined it as a key to quality healthcare, which further increased interest in person-centred care in health policy, healthcare projects and research. The importance of person-centred care has also been rapidly highlighted in the healthcare service sector, as the phenomenon of health consumers seeking quality healthcare becomes salient to economic development [4].

Accordingly, person-centred care has begun occurring in the emergency healthcare environment as well. In the past, the emergency room (ER) was a healthcare service delivery system that was illness- or provider-centred, focusing on speed and efficiency. Recently, several studies have shown a transition of the ER to a venue providing person-centred services that are respectful of patients’ individuality and based on the partnership between patient and medical staff. Person-centred care in the ER is viewed as a key to guaranteeing quality healthcare and promoting patient safety and satisfaction, even in chaotic environments.

Whenever we discuss person-centred care in the emergency room, what is constantly discussed is what person-centred care in the emergency room is and how it can be provided [5] In the ER, overcrowding, chronically inadequate human resource, and frequent patient transfer cause high stress in nurses [6]. Predicting the workload in the ER is impossible because it is common to perform urgent tasks that arise unexpectedly. Additionally, the increased severity of patients’ conditions may result in nurse burnout, beyond high stress. ER problems may seem to weaken nurses’ capability of providing person-centred care and impel them to regard person-centredness as inapplicable to the ER.

Despite such concerns, a study reported that person-centred care increased job satisfaction and reduced burnout in nurses [7]. It is also reported that, with person-centred care, the patients’ ability to care for themselves improved, reducing nurse dependency and decreasing the length of hospital stay, leading to desirable health outcomes, including decreased healthcare cost [8,9,10,11].

Given the changing healthcare environment, it is now time to explore the essence of ER nurses’ person-centred care experiences in depth. The purpose of this study was to understand person-centred care experienced by emergency room nurses in depth and reach at the essence of emergency room nurses’ lived experience of the person-centred care. As the first study of its kind in Korea, the present research was based on in-depth interviews with ER nurses. An understanding of person-centred care will prepare nurses to identify patients’ nursing needs and provide care accordingly, ultimately improving the quality of nursing care in the ER.

## 2. Materials and Methods

### 2.1. Design

In the present qualitative study, ER nurses’ experiences in person-centred care were explored using Colaizzi’s [12] phenomenological method. This was appropriate, as phenomenological research is a qualitative research methodology that seeks to understand complex phenomena through the participants’ lived experience [13]. The Consolidated Criteria for Reporting Qualitative Research (COREQ) reporting guidelines were used in both the framing and reporting of this study to guarantee that sufficient details on the methods of data collection, analysis and interpretation were provided.

### 2.2. Participants

Participants were nurses who had worked for ≥6 months in the ER of a tertiary hospital in B city. The researchers visited the hospitals in B city and posted recruitment flyers on bulletin boards in nurse offices. The flyers contained details of the study’s purposes and procedure; the recruitment of nurses capable of expressing their experiences in concrete terms was announced. Nurses who voluntarily wanted to participate and signed a written consent form were selected. Of them, novices with <6 months experience were excluded because they were considered insufficiently experienced in person-centred care, as their focus would have been getting familiar with the ER job. In total, eight nurses participated.

Participants’ mean clinical experience was 9.1 years, with an ER experience of 7.1 years, on average. In total, five rounds of interviews were conducted. No new information was obtained in the fifth round; thus, it was determined that theoretical saturation was reached.

### 2.3. Data Collection

Data were collected from 15 February to 29 May 2019. It was believed that the interpretation of the term “person-centred care” would vary across participants; therefore, before the first interview, the researcher explained the term as defined in the present study, and instructed them to think exhaustively about their experience of person-centred care. In-depth interviews were conducted in the place and at the time designated by participants, to ensure a quiet and relaxed surrounding.

Interviews began by asking participants about their everyday lives; as the conversation progressed, unstructured questions were asked. Non-verbal communications, such as changes in emotions, intonations, and facial expressions were noted in a notebook. Unstructured questions included the following: ‘Tell me your experience of person-centred care in the ER’, ‘In what aspects is person-centred care in the ER differentiated from that in other departments?’ and ‘What do you think person-centred care in the ER is’?

Three participants worked at the same hospital and began working in the same year. As they were closely acquainted, they were interviewed in a focus-group setting. The remaining five participants were interviewed individually. Sufficient time was given so participants could fully express themselves. Each interview took a minimum of 32 min and a maximum of 70 min. At the end, participants were given a small gift. Interview recordings were transcribed on the same day they were conducted. To ensure data accuracy, whether participants’ wording was accurately transcribed was cross-checked by iteratively listening to the recordings.

### 2.4. Data Analysis

Data were analysed using the phenomenological method proposed by Colaizzi [12], a six-step analytical method to accurately identify meanings from participants’ words and the essence of a phenomenon. It is characterised by re-stating participants’ experiential descriptions into a more general form and describing the fundamental structure of their common experiences.

In the first of the six steps, interview transcriptions were read from beginning to end several times, and interview recordings were repeatedly listened to in order to understand overall meanings in the ER nurses’ experiences of person-centred care. In the second step, the parts in the transcriptions indicating an experience of person-centred care were underlined to extract meaningful statements. One hundred thirty-two meaningful statements pertaining to the core concept of person-centred care were extracted. In the third step, the extracted statements were formulated into more general and abstract statements, and two researchers integrated the general statements conveying similar meanings, while continuously crosschecking with the raw interview data. In the fourth step, similar meanings were grouped together to organise them into themes. During this step, eight themes and four categories were derived. In the fifth step, the ER nurses’ experiences in person-centred care accounted for inclusively by each theme were described and integrated. In the sixth step, their experiences in person-centred care were described using clear statements to identify the fundamental structure of the research issue.

### 2.5. Ethical Considerations

The study was approved by the institutional review board (IRB; H-1902-004-075). After being thoroughly informed of the study purposes, interview process, main questions and interview duration by the researcher, all participants signed a written consent form. They were also informed that they could withdraw from the study at any point in time, that interview recordings and field notes would be completely discarded upon the completion of the research and that they would remain anonymous.

### 2.6. Trustworthiness of the Study

To ensure the trustworthiness of the study, the study was conducted following Lincoln and Guba’s [14] criteria for research trustworthiness, so that study findings would satisfy truth value, consistency, neutrality and applicability to increase reliability and validity. To fully understand the contents of the interviews and obtain reliable data, the interview recordings were repeatedly listened to and cross-checked against the transcriptions. Then, the findings were iteratively compared against the raw interview data to remove any misrepresentation. The results were reviewed by a participant to confirm congruence between participants’ experiences and the results. To reduce between-researcher incongruence, researchers continually exchanged opinions while analysing the data, and congruence was increased by consulting a professor on the research process and findings. To maintain neutrality, the researchers made conscious efforts to exclude preconceptions or biases due to their previous knowledge and experiences. To improve applicability, the researchers tried to interview participants with diverse backgrounds to overcome limitations of relying on experiences that are skewed in a direction and included focus-group interviews to obtain enriched data in a comfortable atmosphere.

## 3. Results

Based on interviews with eight participants, one hundred thirty-two meaningful statements, eight themes, and four categories relating to ER nurses’ experiences in person-centred care were derived. The four categories were ‘an ideal considered difficult to achieve in practice’, ‘the importance of communication’, ‘quantitatively and qualitatively enriched nursing care’ and ‘growth accompanied by reflection’.

### 3.1. Category 1. An Ideal Considered Difficult to Achieve in Practice

Participants said that, as they continued working in the ER, they were gradually becoming ‘machines’ or ‘robots’ who merely worked without any feelings so as to handle the excessive volume of work given the time constraints. Additionally, they said that urgent situations in the ER interfered with the formation of rapport between nurses, patients and guardians, and that inadequate mutual understanding lent itself to superficial relationships in which nurses would be distanced from the patients and guardians. However, they also said that person-centred care was the type of nursing care they were always seeking and would like to provide, if supportable in the ER environment. In this category, the following themes were derived: ‘promptly handling patients in the overcrowded ER is considered as nurses’ fate’ and ‘there should be an appropriate compromise with efficient nursing’.

#### 3.1.1. Theme 1. The Fate of Nurses Handling Promptly Patients in Overcrowded ER

In the situations in which numerous patients are admitted to the ER at a given time, nurses have insufficient time for each patient during their shifts and are unable to listen to them. Participants worried that their colleagues would be burdened with additional work and would then complain because spending more time with one patient would leave less time for other patients. They also mentioned that patients admitted to the ER have physical injuries to be promptly attended to, which takes priority over person-centred care.

*‘Person-centred care would be possible if the ER is not as busy. The longer I work in the ER, the more I feel that patients are my problem to solve, rather than people. I feel like I am gradually moving away from person-centred care. Visible tasks should first be handled, shouldn’t they? So, one could say that person-centred care is being avoided.’*

(RN 5)

*‘Incoming patients don’t have a bed until the saturated number of patients move out, and so, I need to pass them through the ER as maximally fast as possible. Now, the ER is full of patients who cannot lie down because there is no bed.’*

(RN 5)

#### 3.1.2. Theme 2. Necessity of Compromise with Nursing Efficiency

The ideal type of nursing care sought by the participants was close to person-centred care. However, in practice, they were working in a fast turn-around environment and were trying to provide person-centred care within limits.

*‘We are just too busy, and we cannot get to know the patients coming to the ER at every moment. So, we try to provide person-centred care we can give under the condition’.*

(RN 6)

*‘I cannot do much. Person-centred care in the ER would be talking with patients, even if briefly, acknowledging even a small thing about them, saying sympathising words about their anxiety and pain, while I am extremely busy. I think that speaking one word in sympathy would be more effective than saying hundred words’.*

(RN 6)

### 3.2. Category 2. The Importance of Communication

Participants stated that ER patients and their guardians mostly want to know patients’ current condition and the treatments, and that they wait anxiously because emergency treatment is unfamiliar to them. They said that, during such moments, explanations by nurses would be critical, and person-centred care in the ER would be to listen to them and explain what they would like to know before being asked. This category included ‘take the initiative to explain’ and ‘listen to patients’.

#### 3.2.1. Theme 3. Taking the Initiative to Explain

They voiced that taking the initiative to offer explanations to ease patients’ or guardians’ anxiety before they asked would prepare them for the treatment.

*‘There is nothing you can do in the ER except for explaining often. Once provided with an explanation, patients can prepare themselves because now they know what the plan would be. I think explaining to patients and the guardians is the most important because they both could plan ahead for personal businesses to take care of’.*

(RN 7)

*‘Ultimately, the nature of all questions patients ask are similar. When they will be admitted, when test results will be available, how long they need to wait. If I give answers to those questions, it feels like half the work is done’.*

(RN 7)

#### 3.2.2. Theme 4. Listening to Patients

Participants were willing to ask and listen to what patients thought and accept their words, rather than guessing patients and guardians’ emotions and states.

*‘I have created an ER brochure before. I wrote about tests, procedures, and the processes... After completing the brochure, I realised that the conclusion was to be quiet and just wait. I only wrote what I needed to say, and there was no information guardians would probably want to know. I was shocked by the realisation’.*

(RN 7)

*‘It is to listen first to what the other person wants to say, instead of me asking what I want to ask. Patients in the ER have to speak, but it seems that we listen only to what we want to hear and see only what we want to see. We should first listen to what patients want’.*

(RN 8)

### 3.3. Category 3. Quantitatively and Qualitatively Enriched Nursing Care

Participants recalled being busy handling the accumulated tasks due to inadequate knowledge and skills regarding ER nursing and said that professional competence should be the foundation of person-centred care. They expressed that professional competence would include the capabilities of sympathy and communication, and that nurses with such professional competence could provide quantitatively- and qualitatively-enriched nursing care.

#### 3.3.1. Theme 5. Making Efforts to Achieve Professional Competence

Participants mentioned realising that, in person-centred care, professional nursing competence is the foundation through which they could give patients a sense of relief and gain their trust.

*‘You need to have knowledge. Because you can do things for patients as much as you know. Because you can tell them even if it’s just one more thing. You need to know the system to explain to patients. If a patient with fever comes in, surely, blood culture test will be performed and only after the test is done, antibiotics can be used. If you know the flow, you can explain the entire process and handle things at once’.*

(FGD 1)

*‘First, you must sympathise with the patient. What would the patient need? To understand it, you need to be competent. Without competence, you would merely rely on skills. If you are competent, you can see the next step and go, ‘ah, the patient needs this.’ It’s like this’.*

(RN 7)

#### 3.3.2. Theme 6. Not Machine Replaceable

They said, however, that with person-centred care, nurses can provide high-quality nursing care which may not be visible, rather than just focusing on physical clinical indices.

*‘When patient privacy is not protected, patients could feel embarrassed beyond speech or remain scarred psychologically. This is person-centred care. The immediate, visible result may be the same, but there is a qualitative difference. I think it is the same quantitatively, but different qualitatively’.*

(RN 6)

*‘They say that, in the future, everything will be done by machines. But there is one thing a machine cannot replace, and wouldn’t it be person-centred care? Machine is not capable of sympathising’.*

(RN 7)

### 3.4. Category 4. Growth Accompanied by Reflection

As participants recalled their successful person-centred care in the challenging conditions of the ER, they reflected on whether it was due to laziness and rigidity to say that they could not help because they were too busy. Moreover, participants felt empowered and matured from their intense job, through the experience in which ER patients and/or guardians eventually noticed person-centred care and were moved by it, and believed that their way of nursing patients was special.

#### 3.4.1. Theme 7. Making Me Reflect on Myself Who Used to Inertia

While stating that they could not help because they were too busy in the ER or there was inadequate external support, they expressed frustration that the initial enthusiasm toward person-centred care, which they dreamt about when they first decided to be a nurse, was diminishing.

*‘I feel very frustrated when I could not provide person-centred care. I was often argumentative with patients. Yes… I regret when I go home from work. I think ‘I should have been more patient’, and I feel guilty, too’.*

(RN 7)

#### 3.4.2. Theme 8. Feeling like Being Rewarded for the hard Work

Participants experienced job satisfaction, pride and a feeling of being rewarded when patients recovered after they provided person-centred care or when patients and the caregivers were appreciative of them.

*‘I felt rewarded when the patient’s caregiver noticed me and said thank you. I was determined to be sincerer in nursing patients. It felt like I grew more, too’.*

(RN 4)

*‘I clearly felt that patients reacted differently when I provided person-centred care. I speak to them first, and sympathise with them on their moods and feelings. Afterwards, they look for me only. They say thank you. Then, I am proud of myself. I feel pride in performing this job of nursing’.*

(RN 8)

## 4. Discussion

In this study, ER nurses’ person-centred care was explored phenomenologically. The results revealed four categories: ‘an ideal considered difficult to achieve in practice’, ‘the importance of communication’, ‘quantitatively and qualitatively enriched nursing care’ and ‘growth accompanied by reflection’. Unlike other working departments, emergency room nurses recognized that although the environment of emergency room is difficult to achieve person-centred care, successful nursing experience for person-centred care can increase job pride and job satisfaction.

Participants felt pressured to be fast in handling patients in the overcrowded ER, saying that to provide person-centred care in the ER, an appropriate compromise should be made with efficient nursing. This is consistent with previous findings that treatment was often delayed or stopped in the overcrowded ER, and that it negatively affects nursing care by leading nurses to overlook nursing fundamentals [15,16]. Byrne and Heymen [17] also reported that ER nurses were pressured to run the ER smoothly. The nurses were found to allocate much time to efficiently handling healthcare tasks, rather than actually providing nursing care for patients [18,19]. It is reported that nurses regard their role as saving lives, and that most interaction with patients occurs when they perform healthcare tasks or follow doctors’ instructions. In an experimental study on an intervention for person-centred care [20], nurses had a negative perception that person-centred care would require 10–20 min more for each patient. A qualitative study [21] reported that person-centred and non-person-centred moments coexisted, and that provider-centred care generally took priority if there was a need to protect a severe patient, a guardian, or medical staff. Bolster and Manias [22] also reported that, although participants aimed for person-centred care and considered it important, their words and actions were incongruent according to an observation of the participants during drug administration.

The second category was ‘the importance of communication’. Participants stated that ER patients and guardians most wanted to know patients’ current state and the treatment they would receive, and that it is important for nurses not to assume, but ask what patients think and accept what they express. In Walker and Deacon’s [21] study, communication was found to be critical to person-centred care. Participants in that study reported communicating with patients’ families and helping them understand so that they could participate in decision-making and have opportunities to choose. Other studies also mentioned communication with patients as the key in person-centred care [2,23]. Contrarily, in a qualitative study of ICU patients [24], the main attributes identified by the patients were sympathy and patient dignity. It is speculated that, unlike ER patients, ICU patients have already been given the information on their condition and the treatment direction; thus, their need for emotional support remains strong because they are alone, without the accompaniment of the family, in an enclosed space. The ER is a place that patients visit due to various illnesses, sudden pain and accidents, and it is reported that, compared to medical staff, patients perceive their symptom or injury to be more severe and have high anxiety during ER visits [25]. In such a situation, patients want to be emotionally supported with respect to their current condition and information on the tests and processes they will undergo and the expected outcomes [26]. Accordingly, ER nurses should communicate directly with patients to identify their needs, rather than simply guessing, and establish a nursing plan.

The third category was ‘quantitatively and qualitatively enriched nursing care’. Participants said that to provide better person-centred care, they tried to be professionally competent, which also helped them to provide high-quality nursing. In another study, nurses mentioned having a greater need to be equipped with wide-ranging clinical knowledge and to learn interpersonal communication skills to provide person-centred care [27]. In Hong and Kang’s [24] study, too, patients mentioned nurses’ professional competence as an important element. Additionally, other studies argued that person-centred care would guarantee quality healthcare in the chaotic ER [5,28] and have shown powerful evidence that person-centred communication improved clinical outcomes in diabetes, hypertensive and cancer patients [29,30,31,32]. Of the studies in Korea, those with stroke patients, patients with cardiovascular disease [33,34] and women with breast cancer [35] demonstrated that nursing satisfaction increased and depression decreased as the awareness of person-centred care improved, confirming that quantitatively- and qualitatively-enriched nursing is provided through person-centred care.

The fourth category was ‘growth accompanied by reflection’. Participants who had become inert reflected on their experience of successful person-centred care in the arduous ER environment. Positive feedback from patients and guardians enhanced their pride and job satisfaction and induced a feeling of relief from highly intense job stress. Through the experience, participants satisfied their need to further grow as providers of high-quality nursing care. Zempsky and Cravero [36] reported that person-centred care increased job satisfaction in care providers and decreased nurse burnout, again confirming its positive impact. An experimental study comparing a group of nurses providing person-centred care and another providing conventional care found that job satisfaction was higher in the former group [20].

ER nurses’ experiences in person-centred care induced the nurses to believe that person-centred care could be achieved based on communication with patients, through which they could provide high-quality nursing, and enhanced their desire to grow as providers of high-quality nursing. These findings will help nurses better understand person-centred care, despite the existing prejudice that the environment of the ER presents a major barrier to such care. Based on an in-depth understanding of person- centred care experienced by emergency nurses, it will contribute to enhancing the quality of nursing care in the emergency room.

## 5. Conclusions

This qualitative study was conducted to understand the intrinsic meaning and essence of ER nurses’ experiences in person-centred care. The study is significant in that it is the first in Korea that explored the experiences of person-centred care in the ER, where the priority is to perform medical tasks to save lives. The study revealed that the ER nurses tended to regard person-centred care as an ideal difficult to implement in the ER, as their foremost task was to perform tasks that deal with patients’ life and safety in a busy job environment. However, qualitatively and quantitatively enriched experiences of successful nursing care through person-centred care based on communication with patients prompted nurses to reflect on themselves, for they had been disregarding person-centred care as a concept inapplicable in the ER, but having positive impacts, including increasing pride and job satisfaction. Based on the findings, organisational efforts should be made to reduce the dominant conception that person-centred care in the ER is difficult. Positive reinforcement by ER nurses’ experiences in person-centred care will have a diffusion effect such that it would be modelled by nurses in other departments. Such efforts will decrease job stress and increase job satisfaction in nurses, reducing nurse turnover. Additionally, ultimately, patients’ clinical outcomes are expected to improve through high-quality nursing care.

In the future, research should assess the level of person-centred care in the ER to gain a more comprehensive understanding. Moreover, studies should compare person-centred care from the perspectives of patients and nurses and examine the relationship of person-centred care with patient clinical outcomes and/or ER operating indices.

## Data Availability

The data presented in this study are available on request from the corresponding author. The data are not publicly available due to ethical restrictions.
